# Vascular Endothelial Growth Factor -460 C/T BstUI Gene Polymorphism is associated with Primary Open Angle Glaucoma

**DOI:** 10.7603/s40681-014-0004-3

**Published:** 2014-08-06

**Authors:** Hui-Ju Lin, Wen-Lu Chen, Ter-Hsin Chen, Yung-Jen Kung, Lei Wan

**Affiliations:** 1 Department of Ophthalmology, China Medical University Hospital, Taichung, Taiwan; 2 School of Chinese Medicine, China Medical University, No.91 Hsueh-shih Road, 404 Taichung, Taiwan; 3 Graduate Institute of Veterinary Pathobiology, College of Veterinary Medicine, National Chung Hsing University, Taichung, Taiwan; 4 Department of Veterinary Medicine, National Chung Hsing University, Taichung, Taiwan; 5 Department of Biotechnology, Asia University, Taichung, Taiwan; 6 Department of Gynecology, China Medical University Hospital, Taichung, Taiwan

**Keywords:** Primary open angle, Glaucoma, Genetic polymorphism, Vasogenetic endogenous, Growth factor, Nitric oxide, Hypoxia

## Abstract

**Background:**

Hypoxia and nitric oxide (NO) play important roles in the onset and progression of glaucoma. Vascular endothelial growth factor (VEGF) is one of the main factors responsive to hypoxia and NO. In this study, we investigated the association between the *BstU*I C/T VEGF gene polymorphism and primary open angle glaucoma (POAG).

**Methods:**

60 POAG patients and 78 healthy volunteers were enrolled in this study. The most frequently observed polymorphism in the VEGF gene is *BstU*I C/T, which was located 460 nucleotides upstream of the gene. The polymorphism was observed using polymerase chain reaction-based restriction analysis.

**Results:**

Significant differences were observed in the distribution of the polymorphism between control subjects and POAG patients (*p* = 0.003). C/C homozygotes are absent in the control group; therefore, this genotype represents a suitable genetic maker for POAG.

**Conclusions:**

Hypoxia and NO may be involved in the pathway whereby the VEGF-460 polymorphism regulates POAG. Furthermore, homozygous C/C VEGF genotype is a useful maker for Chinese POAG.

**Background:**

Hypoxia and nitric oxide (NO) play important roles in the onset and progression of glaucoma. Vascular endothelial growth factor (VEGF) is one of the main factors responsive to hypoxia and NO. In this study, we investigated the association between the *BstU*I C/T VEGF gene polymorphism and primary open angle glaucoma (POAG).

**Methods:**

60 POAG patients and 78 healthy volunteers were enrolled in this study. The most frequently observed polymorphism in the VEGF gene is *BstU*I C/T, which was located 460 nucleotides upstream of the gene. The polymorphism was observed using polymerase chain reaction-based restriction analysis.

**Results:**

Significant differences were observed in the distribution of the polymorphism between control subjects and POAG patients (*p* = 0.003). C/C homozygotes are absent in the control group; therefore, this genotype represents a suitable genetic maker for POAG

**Conclusions:**

Hypoxia and NO may be involved in the pathway whereby the VEGF-460 polymorphism regulates POAG. Furthermore, homozygous C/C VEGF genotype is a useful maker for Chinese POAG.

## 1. Introduction

Various circulatory abnormalities have been cited as involved in etiology of glaucomatous optic neuropathy.^1^,^2^ Prominent in the disease is vascular regulatory factor itself.^3^ Among vascular factors, recent studies suggest possibility of endothelium-dependent vaso-regulatory system playing a key pathogenetic role.^4^ Ability of cells to sense and respond to changes in oxygen tension is critical for many developmental, physiological, and pathological processes; hypoxia may play a direct role in pathogenesis of optic nerve head cupping, and visual field defects.^4^,^5^ It is evident that hypoxia ranks among the crucial factors in pathogenesis of optic nerve damage and visual field defects in glaucoma. Yancey CM et al. proposed greater intraocular pressure (IOP) leading to decreased choroidal blood flow and outer retinal hypoxia, measured as lesser choroidal pO_2_; this hypoxia is responsible for electroretinogram C-wave^5^.

Nitric oxide (NO) is another vital endothelium-dependent vasoactive mediator acting as a potent vasodilator. Kotiloski H et al. observed that NO concentrations in aqueous humour were slightly higher in glaucoma patients than in controls.^4^ Galassi F et al. also suggested disorders of NO regulatory processes as be involved in modulating blood supply to the optic nerve and in aqueous humour outflow.^1^ Becquet et al. suggested NO acting at the level of ciliary muscle as well as on aqueous outflow pathway (trabecular meshwork, Schlemms’ canal, and collecting channels), thus influencing ocular hydrodynamics.^6^ It is also known that topical or intracameral application of NO donors alter aqueous humour outflow.^7^ Neufeld AH et al. proved induction of nitric-oxide synthesis (NOS-2) in an optic nerve head moderately raising IOP level. Excessive NO production by NOS-2 is cytodestructive to many tissues and neurotoxic to the central nervous system.8 The exact nature of physiopathological interaction between NO vaso-regulation and how it influences aqueous humour outflow remain unclear, but there exists well-established relationship between NO and glaucoma.

Hypoxia modulates expression of a number of genes.^4^,^5^,^9^-^14^ Among these, vascular endothelial growth factor (*VEGF*) gene is viewed as a critical mediator of endothelial sprouting at hypoxic sites.^9^-^14^ It has been proven that NO up-regulates expression of *VEGF*,^12^-^14^ which can be regulated by a variety of stimuli: hypoxia, cobaltous ion, nitric oxide (NO), growth factors, and cytokines.^15^ Hypoxia is regarded as the most potent *VEGF* regulator, and NO has recently drawn much attention as a regulator.^16^,^17^ Hypoxia and NO play important roles in pathogenesis of glaucoma. We correlated VEGF *BstUI* C/T gene polymorphism with primary open angle glaucoma (POAG).

## 2. Materials and Methods

We enrolled POAG patients from the Department of Ophthalmology at China Medical University Hospital from May to July 2003. All patients received serial ophthalmic examination: IOP, visual acuity, gonioscopy, autoperimetry, optic disc examination, and retinal examination. The control group volunteers were selected from patients receiving routine physical examination and were examined by the same ophthalmologist. Volunteers were all free of any systemic disease, patients with ocular disease other than POAG eliminated. POAG patients met at least one of the following criteria. Visual field: 1). At least two abnormal visual field tests by Humphrey automated perimetry, as defined by computer-based objective criteria. 2). Presence of one or more absolute defects in central visual field 30°, with ophthalmologic interpretation as glaucomatous visual field loss. Optic disca: 1). Either horizontal or vertical cup-to-disc ratio 0.6 or more. 2). Narrowest remaining neuroretinal rim of 20% or fewer disc diameters.Ophthalmologic: Patients with other possible causes of disc or field changes other than POAG were excluded.

**Figure 1.**
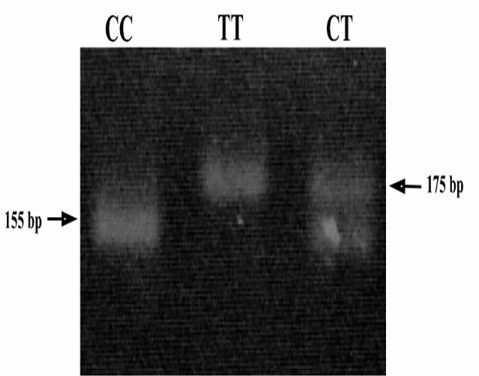
PCR products of VEGF-460 gene C/T polymorphism present on 3% agarose gel. Lane 1 “CC” homozygote: the two *BstUI* cuttable bands were 155 bp and 20 bp. Lane 2 “TT” homozygote: “T” was *BstUI* uncuttable site, and the fragment was measured 175 bp. Lane 3 “TC” heterozygote.

We tabulated VEGF-460 polymorphism in all subjects, comparing its prevalence between control and POAG groups. Odds ratio calculated diverse allele frequencies. This study was conducted out with approval from the Human Study Committee of China Medical College Hospital. Informed consent was obtained from all participants. Genomic DNA was prepared from peripheral blood by Extractor WB kit (Wako, Japan). Polymerase chain reactions (PCRs) for genes were carried out in a total volume of 50*μ*l, containing genomic DNA; 2-6 pmole of each primer; 1X Taq polymerase buffer (1.5 mM MgCl_2_); and 0.25 units of AmpliTaq DNA polymerase (Perkin Elmer, Foster City, CA). Primers for genes are forward 5’-TGTGCGTGTGGGGTTGAGCG-3’and reverse 5’-TACGT GCGGACAGGGCCTGA-3’ according to Watson et al.2 PCR amplification used programmable thermal cycler GeneAmp PCR System 2400 (Perkin Elmer). The 175 bp PCR product was mixed with 2 units of *Bst*U I (Takara, Japan) and the reaction buffer as per manufacturer’s instructions. Restriction site was located -460 bp upstream of exon I, (C to T); transcription site “C” was cuttable. Two fragments measuring 155 bp and 20 bp were present if product was digestable. Uncuttable band was 175 bp on gel. Reaction was incubated for 2 hours at 37°C, 10 βl of the products loaded onto a 3% agarose gel containing ethidium bromide for electrophoresis. Polymorphism was categorized as “TT” (cuttable) and “CC” (uncuttable) homozygote or “TC” heterozygote (Figure 1).

For statistical analysis of the allelic frequency distribution in this polymorphism, both groups were compared using the chi-square test. The software used for the calculation was the SPSS® system. When the assumption of the chi-square test was violated (i.e. when 1 cell had an expected count of <1, or > 20% of the cells had an expected count of <5), the Fisher’s exact test was performed. A *p*-value of <0.05 was considered statistically significant.

## 3. Results

The POAG group consisted of 60 patients, the control group 78 healthy volunteers. The latter ranged in age from 22 to 70 (mean: 53) years and had no ophthalmic disease, all Chinese and unrelated, including 40 women and 38 men. POAG patients ranged in age from 20 to 70 (mean: 48) years, were unrelated, including 30 women and 30 men. All patients were followed up two to eight (average of five) years. Ten received trabeculectomy, two of these at two different sites; fifty used topical drugs to control intraocular pressure. Each patient used an average of 1.3 types of anti-glaucomatous drugs. Ten patients required no drugs to control IOP following trabeculectomy. Table 1 plots VEGF genotype frequencies between groups: significant difference appears in C/C polymorphism distribution between normal controls and POAG patients (*p* = 0.003). Distribution in the POAG group was “T/T” homozygote, 43.3%; “C/T” heterozygote, 43.3%; and “C/C” homozygote, 13.4%. Distribution of CC homozygote was sharply higher in the POAG group.

**Table 1.** Distribution of VEGF gene –460 *BstUI* polymorphism between healthy control subjects and POAG patients exam by Fisher’s exact test

	TT	TC	CC	Total	*p*-valve
Control	38 (48.7%)	40 (51.3%)	0 (0%)	78 (100.0%)	0.003
POAG	26 (43.3%)	26 (43.3%)	8 (13.4%)	60 (100.0%)	

Furthermore, odds ratio of C/C genotype was significant when analyzed, using regression method according to age (Table 2). This indicates age as an insignificant source of variation. We also calculated “power” of the test of the null hypothesis by SPSS^R^ and found a power of 89% to yield

**Table 2.** Age-adjusted tests for genotypes of VEGF-460 gene polymorphism

Genotype	Odds ratio (95% CI)^+^	
	Unadjusted	Age-adjusted
C/C	1^*^ (2.3-24.60	8.5^*^ (2.4-29.5)
C/T	1	1
T/T	1.4^*^(0.8-2.6)	1.8^*^(0.9-3.5)

^+^CI denotes confidence interval. ^*^ *p*<0.05

## 4. Discussion

Hypoxia and NO play pivotal roles in POAG pathogenesis. Regardless, VEGF appears as a key factor in POAG development. Ankeno N et al. demonstrated hypoxia transcriptionally activating VEGF expression by elevating level of basic helix-loop-helix (bHLH)-PAS transcription factor.^13^ We suspect hypoxia inducing optic axon apoptosis, possibly influenced by altering VEGF transcription. Kimura H et al. proposed NO and hypoxic pathways of VEGF induction sharing common traits and NO mediating transcription by a mechanism distinct from hypoxia.^14^ Regardless of pathways, the fact that hypoxia and NO activate VEGF transcription suggests expression of VEGF as closely related to POAG. Yet VEGF-induced angiogenesis might not be the primary factor in this relationship. VEGF is a cytokine, and our laboratory has demonstrated POAG’s positive association with many cytokines: e.g., tumor necrosis factor alpha^18^ and interleukin-1.^19^ Pharmacological neuroprotection via inhibition of NO and hypoxia may prove useful in treating glaucoma.^5^,^6^

We suspected that VEGF polymorphisms render optic nerve axons less capable of tolerating hypoxic and NO damage and predispose POAG patients to optic neuropathy. Excluding effects of hypoxia and NO, POAG is characterized by optic nerve death, known as mediated by apoptosis.^19^,^20^ VEGF may also alter apoptotic conditions in optic axons through autocrine and paracrine mechanisms, yielding further evidence of a connection between POAG and VEGF, but we do not claim VEGF as direct cause. We suggest an association between them. Precise effect of VEGF on POAG is unknown and may warrant resolution via future use of “proteomic” analysis to identify factors as critical to pathological mechanisms. Our study noted starkly different distribution of VEGF-460 polymorphism between control and POAG groups. Ratio of C/C homozygote is 0% in controls, indicating C/C homozygote as useful for predicting POAG. Association of single nucleotide polymorphism (SNPs) with disease does not indicate genes as directly causes. It should be understood that there is just correlation, while environment effects and post translation may allow full understanding of pathology. We probe candidate genes in order to determine their role in POAG. Likewise, only a fraction of SNPs of these genes were examined; we selected POAG patients who meet the criteria for this study. We can say that VEGF -460 gene polymorphisms are linked with Chinese POAG and can identify Chinese POAG patients. Still, we cannot say that polymorphisms worsen prognosis or alter drug response. Our laboratory has linked certain SNPs with POAG.^17^,^21^ SNPs hold vital implications for human genetics. Understanding associated polymorphism is expected to lend insight into course and treatment of disease. Our ongoing studies correlate SNPs with glaucoma, using SNPs to map Chinese POAG.

## Acknowledgments:

This study was funded by a grant (DMR-103-066) from China Medical University Hospital.
